# Supplementary material for: Optogenetic control of PLC-γ1 activity polarizes cell motility
Source: bioRxiv. 2025 Oct 11:2025.10.09.681531. Preprint. [Version 1] doi: 10.1101/2025.10.09.681531 (PMC12632280; doi:10.1101/2025.10.09.681531)
Supplement: Supplement 7 [file NIHPP2025.10.09.681531v1-supplement-7.pdf]

bioRxiv preprint doi: https://doi.org/10.1101/2025.10.09.681531; this version posted October 11, 2025. The copyright holder for this preprint (which was not certified by peer review) is the author/funder, who has granted bioRxiv a license to display the preprint in perpetuity. It is made available under aCC-BY 4.0 International license.

SUPPLEMENTAL FIGURES

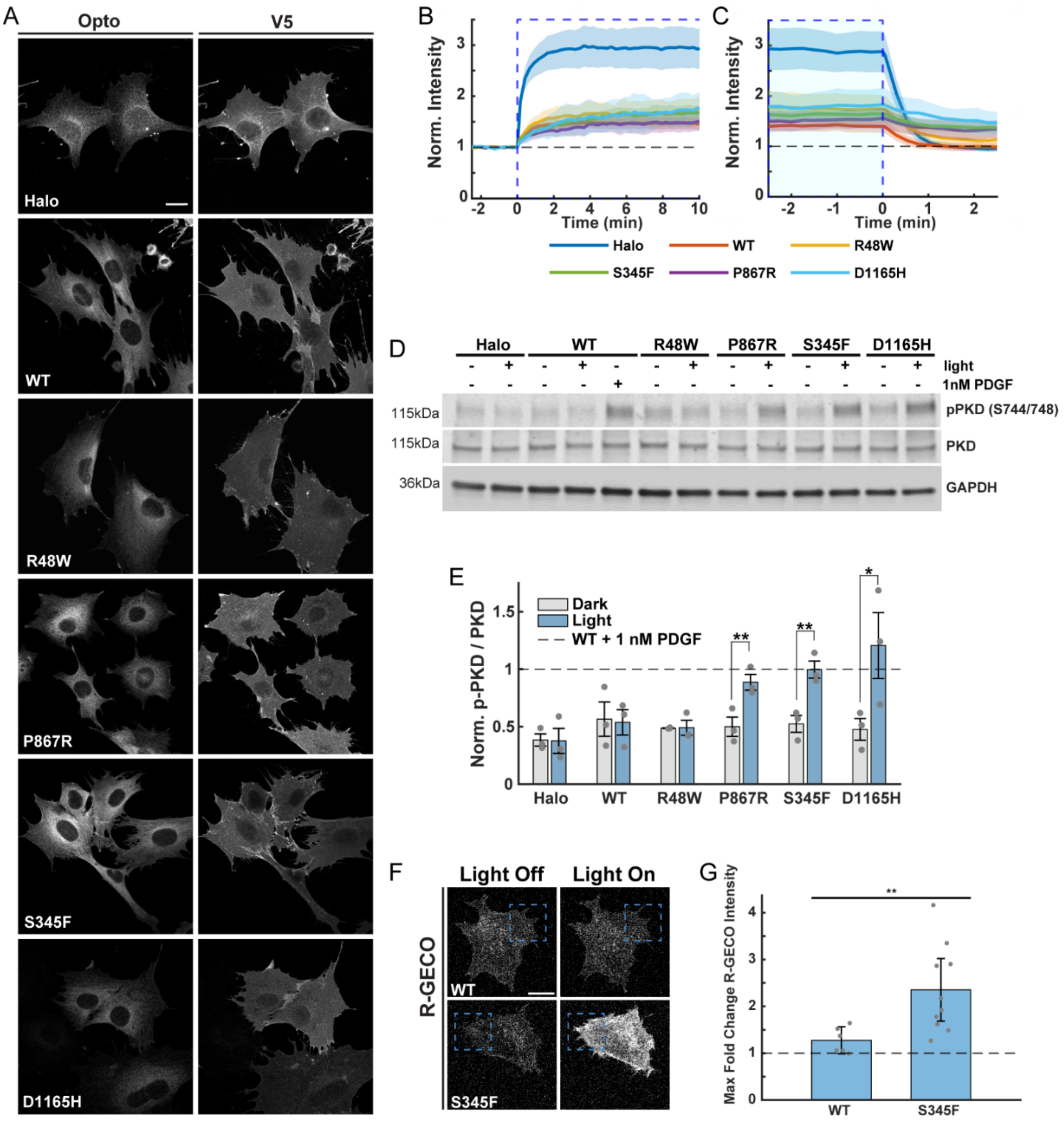

Figure S1: Inducible expression of OptoPLC-γ1 enables spatiotemporal control of membrane recruitment and enzyme activity. (A) Confocal, fluorescence micrographs of fixed, non-stimulated *Plcg1*-null fibroblasts induced to express V5-iLID-CaaX and Halo-SspBμ or PLCγ1-Halo-SspBμ variants. Cells were labeled with JF646 and

bioRxiv preprint doi: https://doi.org/10.1101/2025.10.09.681531; this version posted October 11, 2025. The copyright holder for this preprint (which was not certified by peer review) is the author/funder, who has granted bioRxiv a license to display the preprint in perpetuity. It is made available under aCC-BY 4.0 International license.

immunostained for V5. **(B-C)** Time-course of Halo-JF646 enrichment within the photoactivated ROI in *Plcg1*-null fibroblasts rescued with the indicated OptoHalo/OptoPLC-γ1 variants upon focal illumination with a 488 nm laser. The time axes in (B) and (C) are scaled relative to the start and stop of focal illumination, respectively. Solid, colored lines represent the mean of the respective intensities for OptoHalo (n = 22 cells), OptoPLC-γ1 WT (n = 26 cells), OptoPLC-γ1 R48W (n = 5 cells), OptoPLC-γ1 P867R (n = 5 cells), OptoPLC-γ1 S345F (n = 25 cells), and OptoPLC-γ1 D1165H (n = 6 cells) and the shaded band regions indicate the 95% confidence interval. **(D)** Immunoblot and **(E)** quantification of PKD and associated Ser744/748 phosphorylation in *Plcg1*-null fibroblasts rescued with either OptoHalo or OptoPLC-γ1 variants and globally stimulated with blue light, or 1 nM PDGF, for 5 minutes. Induction was performed by treating with 500 ng/ml doxycycline for 48 hours prior to stimulation. Blot is representative of n = 3 biological replicates. Phosphorylated S744/748 data are first normalized by total PKD and then by the p-PKD/PKD ratio of the WT OptoPLC-γ1 treated with 1 nM PDGF. The normalized data are reported as the mean. To test if normalized p-PKD increased upon light stimulation, dark vs. light treatments for each Opto variant were compared using a paired one-sided t-test. *$p<0.05$ and **$p<0.01$. **(F)** Representative live-cell confocal micrographs of the calcium biosensor R-GECO expressed in *Plcg1*-null fibroblasts rescued with OptoPLC-γ1 WT (n = 6 cells) or OptoPLC-γ1 S345F (n = 10 cells) and focally photoactivated. Dashed, blue box indicates region of interest (ROI) illuminated with a 488 nm laser. **(G)** Maximum fold change, relative to pre-photoactivation baseline, of whole-cell R-GECO fluorescence intensity observed up to 40 seconds after photoactivation. Data are reported as the mean ± 95% confidence interval. WT and S345F were compared using a two-sided Welch's t-test. **$p<0.01$.

bioRxiv preprint doi: https://doi.org/10.1101/2025.10.09.681531; this version posted October 11, 2025. The copyright holder for this preprint (which was not certified by peer review) is the author/funder, who has granted bioRxiv a license to display the preprint in perpetuity. It is made available under aCC-BY 4.0 International license.

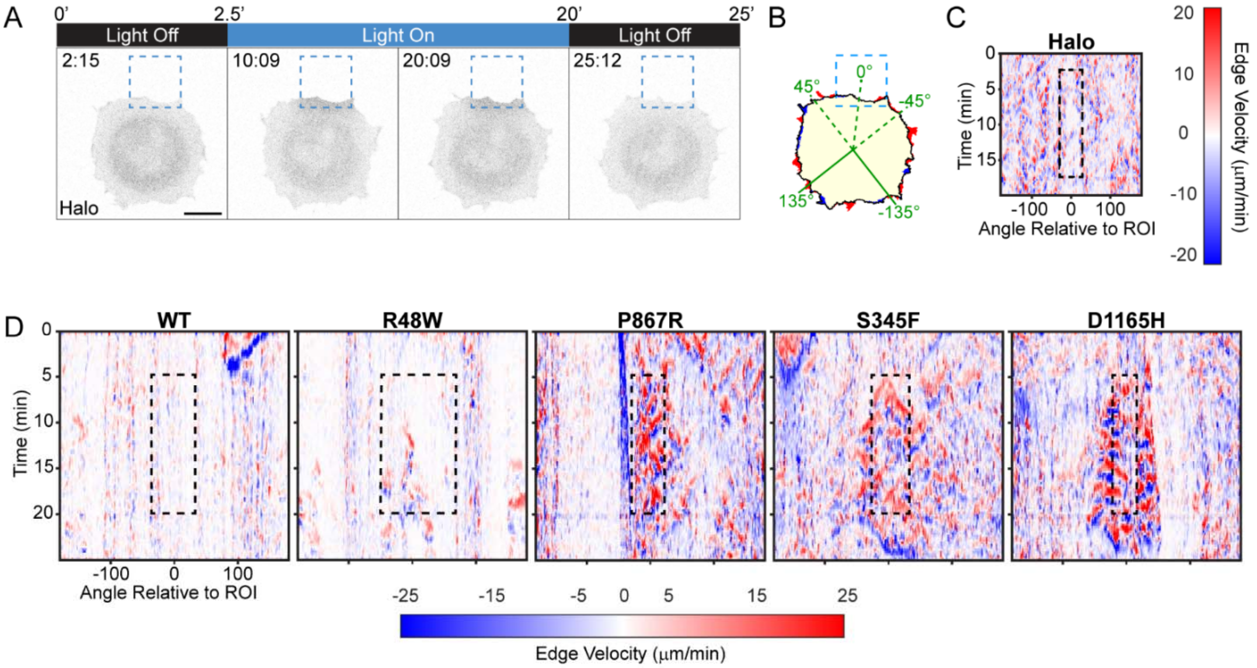

Figure S2: **Photoactivating S345F produces protrusion within and proximal to the illuminated ROI and retraction on the opposing side of the cell. (A)** Time-lapse confocal micrographs of *Plcg1*-null fibroblasts expressing OptoHalo labeled with JF646 ligand, and photoactivated as indicated. Dashed, blue box indicates ROI illuminated with a 488 nm laser. Time indicates min:sec. **(B)** Net protrusion (red) and retraction (blue) for the representative cell shown in **A**. **(C)** Spatiotemporal map of edge velocity prior to, during, and after photoactivation for the representative OptoHalo cell shown in **A**. Zero degrees is defined by the line linking the centroid of the ROI and the centroid of the cell immediately prior to photoactivation (see pixel map in **B**). The dashed, black box denotes the spatiotemporal position of the illuminated ROI. **(D)** Spatiotemporal map of edge velocity prior to, during, and after photoactivation for the representative OptoPLC-γ1 variants shown in **Fig. 2A&B**. Here and hereafter, the dashed, black box denotes the spatiotemporal position of the ROI at the time of photoactivation.

bioRxiv preprint doi: https://doi.org/10.1101/2025.10.09.681531; this version posted October 11, 2025. The copyright holder for this preprint (which was not certified by peer review) is the author/funder, who has granted bioRxiv a license to display the preprint in perpetuity. It is made available under aCC-BY 4.0 International license.

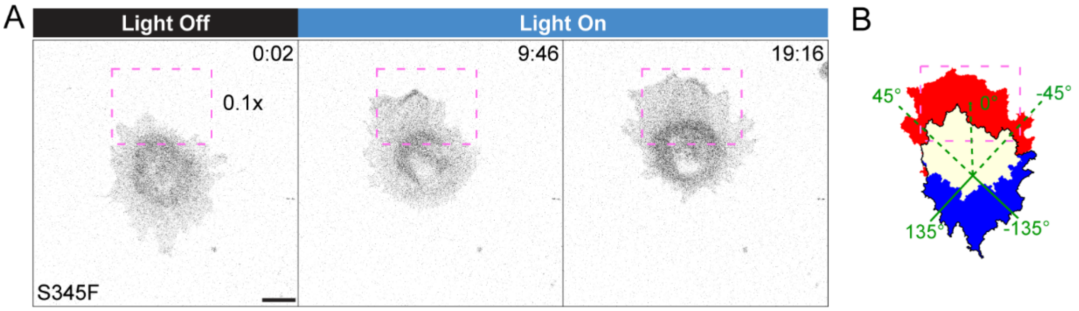

Figure S3: S345F photoactivation at 0.1x relative power polarizes cell motility. (A) Time-lapse confocal micrographs of an OptoPLC-γ1 S345F cell labeled with JFX554 ligand, photoactivated as indicated. Dashed, pink box indicates ROI illuminated with a 488-nm laser set to 0.01% (0.1x) power. Time indicates min:sec, and scale bar = 20 µm. **(B)** Net protrusion (red) and retraction (blue) for the representative cell shown in **A**.

bioRxiv preprint doi: https://doi.org/10.1101/2025.10.09.681531; this version posted October 11, 2025. The copyright holder for this preprint (which was not certified by peer review) is the author/funder, who has granted bioRxiv a license to display the preprint in perpetuity. It is made available under aCC-BY 4.0 International license.

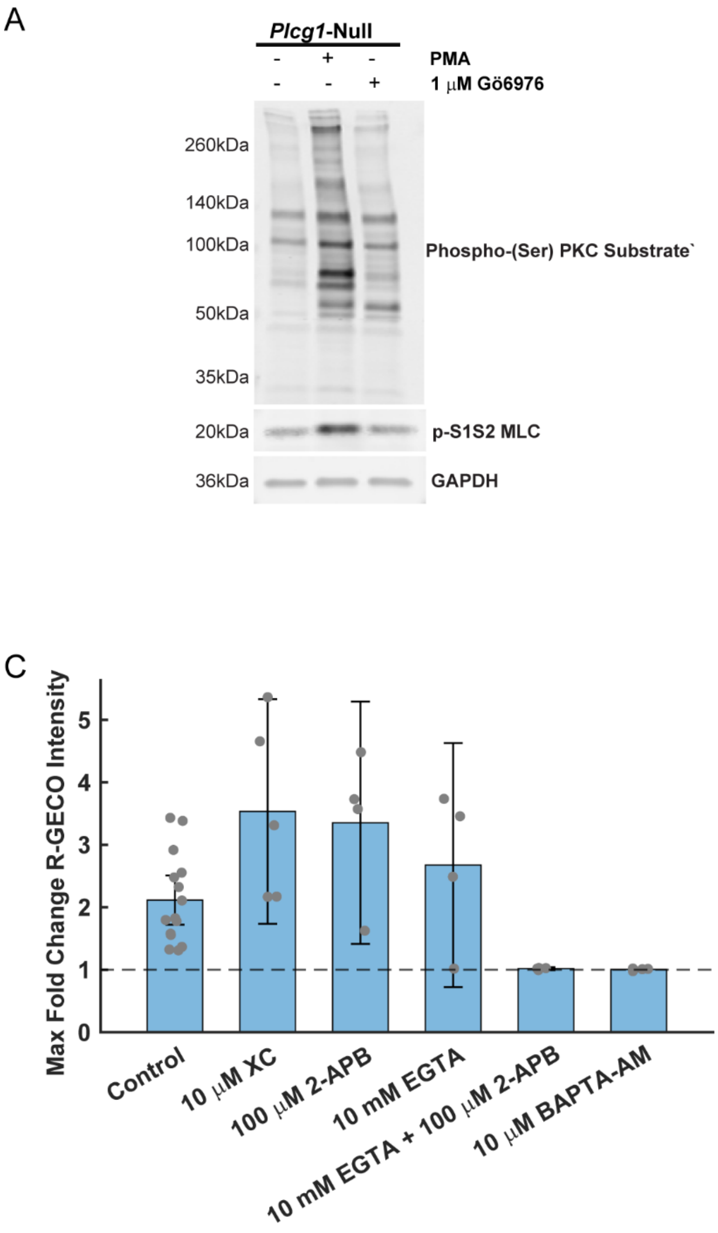

Figure S4: Validation of pharmacological inhibition of PKCα and calcium signaling. (A) Immunoblot of serum-starved *Plcg1*-null fibroblasts stimulated for 5 minutes with 200 nM PMA in the presence of either a vehicle control (0.1% DMSO) or G□6976 and subsequently blotted with antibodies against phospho-(ser) PKC substrate and phosphorylated Ser1Ser2 myosin light chain (MLC). Blot shows the result one biological replicate. **(B)** Representative live-cell confocal micrographs of the calcium biosensor R-GECO expressed in *Plcg1*-null fibroblasts rescued with OptoPLC-γ1 S345F and focally photoactivated without or in the presence of the listed treatments (control: n

bioRxiv preprint doi: https://doi.org/10.1101/2025.10.09.681531; this version posted October 11, 2025. The copyright holder for this preprint (which was not certified by peer review) is the author/funder, who has granted bioRxiv a license to display the preprint in perpetuity. It is made available under aCC-BY 4.0 International license.

=15 cells, 10 µM XC: n = 5 cells, 100 µM 2-APB: n = 4 cells, 10 mM EGTA: n = 4 cells, 10 mM EGTA + 100 µM 2-APB: n = 4 cells, 10 µM BAPTA-AM: n = 4 cells). Dashed, blue box indicates region of interest (ROI) illuminated with a 488 nm laser. **(C)** Maximum fold change, relative to pre-photoactivation baseline, of whole-cell R-GECO fluorescence intensity observed up to 40 seconds after photoactivation. Data are reported as the mean ± 95% confidence interval.

bioRxiv preprint doi: https://doi.org/10.1101/2025.10.09.681531; this version posted October 11, 2025. The copyright holder for this preprint (which was not certified by peer review) is the author/funder, who has granted bioRxiv a license to display the preprint in perpetuity. It is made available under aCC-BY 4.0 International license.

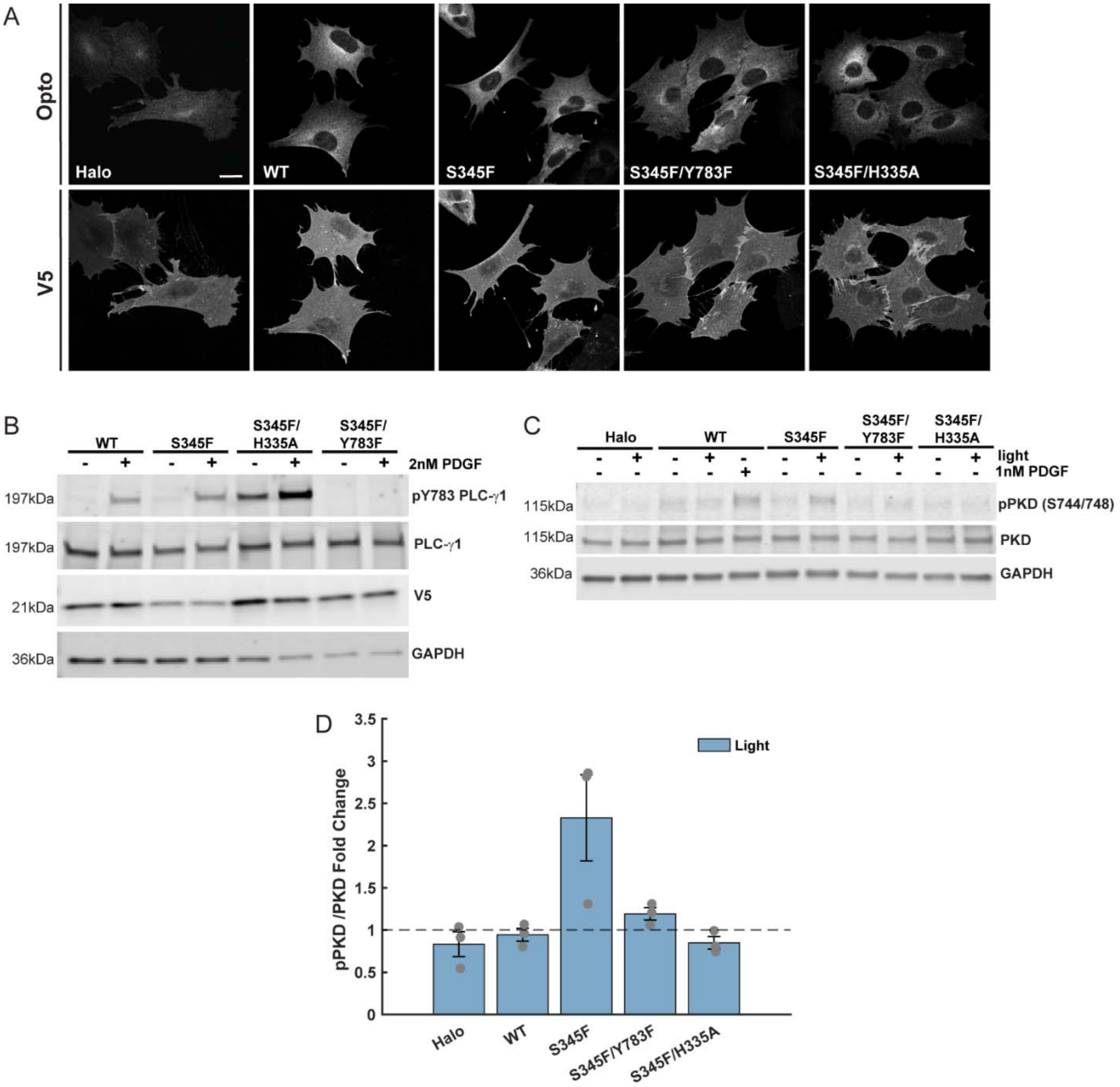

Figure S5: OptoPLC-γ1 S345F double-mutants are inducibly expressed with comparable V5/Halo ratios as WT and S345F variants. **(A)** Confocal, fluorescence micrographs of fixed, non-stimulated *Plcg1*-null fibroblasts induced to express V5-iLID-CaaX and Halo-SspBμ or PLCγ1-Halo-SspBμ variants. Cells were labeled with JF646 and immunostained for V5. **(B)** Immunoblot of PLC-γ1 and associated Tyr783 phosphorylation in *Plcg1*-null fibroblasts rescued with the listed OptoPLC variants and stimulated with 2 nM PDGF, for 5 minutes. Induction was performed by treating with 500 ng/ml doxycycline for 48 hours prior to stimulation. Blot is representative of a single

bioRxiv preprint doi: https://doi.org/10.1101/2025.10.09.681531; this version posted October 11, 2025. The copyright holder for this preprint (which was not certified by peer review) is the author/funder, who has granted bioRxiv a license to display the preprint in perpetuity. It is made available under aCC-BY 4.0 International license.

biological replicate. **(C)** Immunoblot of PKD and associated Ser744/748 phosphorylation in *Plcg1*-null fibroblasts rescued with either OptoHalo or OptoPLC variants and globally stimulated with blue light, or 1 nM PDGF, for 5 minutes. Induction was performed by treating with 500 ng/ml doxycycline for 48 hours prior to stimulation. Blot is representative of n = 3 biological replicates. **(D)** Immunoblot quantification of PKD S744/748 phosphorylation across OptoHalo/OptoPLC variants globally stimulated with light for 5 minutes. Total and phospho-ser 744/748 PKD are first normalized by their associated GAPDH loading controls before calculating the ratio of phospho-PKD to total PKD. The light condition is then normalized by the dark state for each variant. Data reported as the mean ±95% confidence interval.

bioRxiv preprint doi: https://doi.org/10.1101/2025.10.09.681531; this version posted October 11, 2025. The copyright holder for this preprint (which was not certified by peer review) is the author/funder, who has granted bioRxiv a license to display the preprint in perpetuity. It is made available under aCC-BY 4.0 International license.

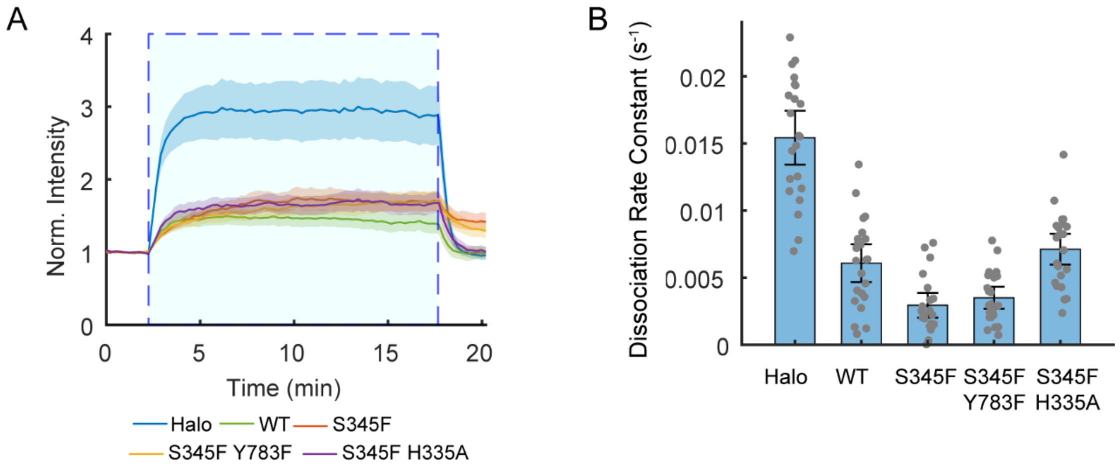

Figure S6: OptoPLC-γ1 S345F double-mutants are enriched upon focal photoactivation. **(A)** Time-course of Halo-JF646 enrichment within the photoactivated ROI in *Plcg1*-null fibroblasts rescued with the indicated OptoHalo/OptoPLC-γ1 variants upon focal illumination with a 488 nm laser. Solid, colored lines represent the mean of the respective intensities for OptoHalo (n = 22 cells), OptoPLC-γ1 WT (n = 23 cells), OptoPLC-γ1 S345F (n = 22 cells), OptoPLC-γ1 S345F/Y783F (n = 23 cells), and OptoPLC-γ1 S345F/H335A (n = 24 cells). The shaded band regions indicate the 95% confidence interval. **(B)** Estimated decay rate constants upon stopping focal activation (see Materials and Methods). Data are presented as the mean ± 95% confidence interval.
